# Incidence and Risk Factors of Early-onset Glaucoma following Pediatric Cataract Surgery in Egyptian Children: One-year Study

**DOI:** 10.5005/jp-journals-10028-1229

**Published:** 2017-10-27

**Authors:** Ghada I Gawdat, Maha M Youssef, Nermeen M Bahgat, Dina M Elfayoumi, Mohamed AS Eddin

**Affiliations:** 1 Professor, Department of Ophthalmology, Faculty of Medicine, Cairo University, Giza, Egypt; 2 Lecturer, Department of Ophthalmology, Faculty of Medicine, Cairo University, Giza, Egypt; 3 Lecturer, Department of Ophthalmology, Faculty of Medicine, Cairo University, Giza, Egypt; 4 Assistant Professor, Department of Ophthalmology, Faculty of Medicine, Cairo University, Giza, Egypt; 5 Assistant Professor, Department of Ophthalmology, Faculty of Medicine, Cairo University, Giza, Egypt

**Keywords:** Aphakic glaucoma, Congenital cataract, Incidence, Prospective, Risk factors.

## Abstract

**Aim:**

To study the incidence and risk factors of glaucoma occurring within 1 year following pediatric cataract surgery in Egyptian children.

**Materials and methods:**

This is a prospective nonrandomized study conducted at Aburich Children’s Hospital, over a period of 1 year on a cohort of Egyptian patients with congenital and infantile cataract. One hundred and fifty eyes of 88 patients were enrolled in this study. All the patients underwent anterior approach removal of lens matter, whereas primary intraocular lens (IOL) implantation was carried at the age of 1 and 2 years for unilateral and bilateral cases respectively. Intraocular pressure (IOP) was measured at 1 week, 1 month, 3 months, 6 months, 9 months, and 1 year. For those who developed glaucoma, time of diagnosis and associated risk factors were reported.

**Results:**

The incidence of glaucoma was 11.33% (17 of 150 eyes), while incidence of glaucoma suspect was 0.67% (1 of 150 eyes) in the first year following cataract surgery. The majority of the cases (66.7%) were discovered in the first 3 months postcataract surgery. Age at time of cataract surgery, the state of aphakia/pseudophakia, persistent fetal vasculature (PFV), and microphthalmia were not found to be significant predictors of early-onset glaucoma in our study.

**Conclusion:**

Aphakic glaucoma continues to be a devastating condition with high incidence during first year following cataract surgery.

**Clinical significance:**

Regular follow-up should start as early as possible following cataract surgery. Further prospective studies with larger study population are required.

**How to cite this article:** Gawdat GI, Youssef MM, Bahgat NM, Elfayoumi DM, Eddin MAS. Incidence and Risk Factors of Early-onset Glaucoma following Pediatric Cataract Surgery in Egyptian Children: One-year Study. J Curr Glaucoma Pract 2017;11(3):80-85.

## INTRODUCTION

Glaucoma is one of the most severe, vision-threatening complications of congenital cataract surgery. The incidence has been variably reported between 6 and 75.9% in the literature.^1-4^

Similarly, the risk factors have been variably reported too. Surgery at a very early age,^5-7^ microphthalmia,^8,9^ and PFV^7,10^ have been implicated in some reports. Some studies even find primary IOL implantation protective,^6,11^ whereas others do not.^10^

Earlier studies have shown that the onset of secondary glaucoma following cataract surgery is usually years after the cataract surgery.^12-15^ Later, it was suggested that glaucoma onset may be earlier than previously suggested.^10,16,17^

So, we conducted this study to determine the incidence and risk factors associated with secondary glaucoma occurring within 1 year following pediatric cataract surgery, in an Egyptian cohort of children from Aburich hospital, over a 1-year period.

## MATERIALS AND METHODS

This is a prospective nonrandomized study, carried out on a cohort of Egyptian patients with congenital and infantile cataract. Patients were selected from the Ophthalmology outpatient clinic of Aburich children hospital, over a period of 1 year: From Jan 2012 to Dec 2012. Aburich hospital is a central University hospital in Cairo and it is the center for referral from most of the governorates in Egypt.

Approval for the study was obtained from the Ophthalmology Department’s ethical committee (according to the World Medical Association Declaration of Helsinki). All patients’ parents or guardians received a thorough explanation of the study design and aims, and were provided with written informed consent.

One-hundred and fifty eyes among 88 patients, of which 42 were females (47.7%), were enrolled in this study. Cases of PFV not associated with visible stretching of ciliary processes or involvement of retina and/or the optic nerve and patients with microphthalmia without any other structural abnormality other than cataract were included to assess these conditions as possible risk factors of glaucoma.

Patients excluded from our study were those with preexisting glaucoma, history of trauma, serious ocular malformation, uveitis, and subluxation. Patients with planned primary IOL implantation who were left aphakic due to intraoperative complications were excluded as well. Similarly younger patients where primary IOL implantation was not planned and developed complications that may lead to increased IOP postoperatively, such as dropped cortical matter, were not included in the study.

### Preoperative Examination

A full ophthalmological history was taken. Age at time of surgery, sex, axial length, laterality, associated systemic and ocular anomalies were recorded.

Slit-lamp examination of the anterior segment was performed in cooperative children. Intraocular pressure was measured using the Perkins hand-held applanation tonometer (Perkins Tonometer Mk2, Clement Clarke International, Edinburgh Way, England).

Ultrasonography of the posterior segment was resorted to whenever a detailed fundus examination was not feasible due to media opacities, and also to measure the axial length.

### Operative Procedure

Removal of lens was performed through two limbal incisions. It is either in the form of lensectomy using automated vitrectomy probe or bimanual irrigation/ aspiration, followed by posterior capsulotomy and limited anterior vitrectomy (for those <6 years old). We do not use triamcinolone for anterior vitrectomy. Primary IOL implantation was carried out at the age of 1 year for unilateral cataracts and 2 years for bilateral cases. Otherwise, patients were left aphakic waiting for proper timing of secondary implantation. In cases of primary IOL implantation, a foldable IOL (AcrySof single piece IOL, Alcon laboratories, Inc., Fort Worth, USA) was placed in the bag, while in cases of secondary implantation AcrySof multi piece IOL (Alcon laboratories, Inc., Fort Worth, USA) was implanted in the sulcus. Postoperative topical dexamethasone 0.1% or prednisolone acetate 1% drops, topical antibiotics, and topical cycloplegic eye drops were given for 4 to 6 weeks.

### Postoperative Examination and Follow-up

The IOP was measured at 1 week, 1 month, 3 months, 6 months, 9 months, and 1 year. Some patients continued to have their IOP checked at 2 and 3 years after surgery. For younger children, IOP was measured under chloral hydrate sedation (50 mg/kg). Refraction and fundus examination were done at 1, 3, 6, and 12 months postoperatively.

We adopted the criteria of Infant Aphakia Treatment Study (IATS)^10^ for diagnosis of glaucoma. Diagnosis of glaucoma was made when the IOP was >21 mm Hg with one or more of following (progressive optic disk cupping, increase in horizontal corneal diameter, increased myopic shift, and the use of surgery to control IOP). The diagnosis of glaucoma suspect was considered when the IOP was found to be >21 mm Hg on two different occasions after topical steroids had been stopped or glaucoma medication use for IOP control without any of the anatomical changes mentioned above.

For those diagnosed as glaucoma, time at which diagnosis was made, and associated risk factors, namely the age at time of surgery, associated ocular anomalies, and aphakia/pseudophakia status of the eye, were reported.

Gonioscopic features were recorded for patients. Some of them had their angles further assessed by anterior segment optical coherence tomography (RTVue; Optovue 100, Inc, Fremont, CA) and ultrasound biomicroscopy (UBM Plus, Model P 45, Paradigm) for documentation.

### Statistical Analysis

Data were statistically described in terms of mean ± standard deviation (SD), median and range, or frequencies (number of cases) and percentages when appropriate. Comparison of numerical variables between the study groups was done using Student’s t-test for independent samples in comparing two groups, and paired t-test for dependent samples. For comparing categorical data, chi square test was performed. Exact test was used instead when the expected frequency is less than 5. Demographic, ocular, and systemic predictors of incident glaucoma were examined using Cox proportional hazards regression to generate hazard ratios, associated 95% confidence intervals (CIs), and relative risk (RR) and also using multiple linear regression analysis to study their effect on postoperative glaucoma. All p-values less than 0.05 were considered statistically significant. All statistical calculations were done using Statistical Package for the Social Sciences (SPSS Inc., Chicago, Illinois, USA) version 18 for Microsoft Windows.

## RESULTS

### Patient Data

Of these 88 patients, 26 had a unilateral cataract (29.5%), and 62 had bilateral cataract (70.5%).

Eighteen patients (12%) had systemic disorders as follows: 9 (50%) patients had cerebral palsy (CP), 2 (11.1%) had delayed milestones, 2 (11.1%) patients were diagnosed as Down Syndrome, 1 (5.6%) patient had galactosemia, 2 (11.1%) patients had hypocalcemia, and 2 (11.1%) patients had renal disease.

Twenty eyes (13.3%) had ocular anomalies in the form of PFV (25%), microphthalmia (65%), and albinism (10%).

### Preoperative Data

The preoperative IOP ranged from 8 to 18 mm Hg with a mean of 10.97 ± 1.68 mm Hg and the axial length measurement ranged from 15 to 25 mm with a mean of 19.01 ± 2.329 mm.

### Operative Data

The mean age of those children at the time of cataract surgery was 17.78 ± 22.73 months with a range of 1 to 153 months. When the age at cataract surgery was divided into two groups, 59.3% eyes were operated before or at the age of 12 months while 40.7% were operated afterward.

One-hundred and nine eyes (72.66%) had lensectomy, while 41 eyes (27.3%) had bimanual irrigation/aspiration with primary IOL implantation.

Also 21 eyes (14%) had secondary IOL implantation and 8 more eyes (5.3%) had a secondary procedure in the form of membranectomy in the first postoperative year.

### Postoperative Data

Eighty-eight eyes (58.67%) were aphakic, while 62 eyes (41.3%) were pseudophakic (whether as a primary procedure or secondary IOL implantation).

### Postoperative Glaucoma

The incidence of glaucoma was 11.3% (17 of 150 eyes), while incidence of glaucoma suspect was 0.67% (1 of 150 eyes), with a total of 12% developing glaucoma-related adverse event, in the first year following cataract surgery. The one eye that has been diagnosed as glaucoma suspect has been included in statistical analysis together with the eyes that have developed glaucoma, being very small in number to constitute a separate group, and they were all referred to as glaucoma-related adverse event. The interval between the cataract surgery and the diagnosis of glaucoma-related adverse events varied from 1 week to 1 year. The majority of the cases (66.7%) were discovered in the first 3 months postcataract surgery (Table 1).

**Table 1:** **Table 1:** Timing of glaucoma diagnosis postcataract surgery

*Timing of glaucoma diagnosis postcataract surgery*		*No of eyes (%) (n = 18)*		*Maximum IOP recorded (mm Hg)*	
1 week		1 (5.6%)		25	
1 month		8 (44.4%)		30	
3 months		3 (16.7%)		30	
9 months		4 (22.2%)		28	
12 months		2 (11.1%)		28	

Glaucoma-related adverse event was diagnosed at a mean age of 17.94 ± 23.29 months, and 4.40 ± 4.45 months from cataract surgery.

Sixty-six eyes (44%) completed follow-up for 2 years and 6 eyes (4%) completed follow-up for 3 years. Two eyes developed glaucoma in the second year following surgery. However, these data had not been analyzed statistically due to two reasons. First, there was a large number of dropouts. Second, we were mainly interested in early-onset glaucoma occurring in first year following surgery as we noticed that some patients developed glaucoma that early in our institute and this triggered our interest in designing such a study.

### Gonioscopic Features of Glaucomatous Eyes

**Figs 1A and B:**
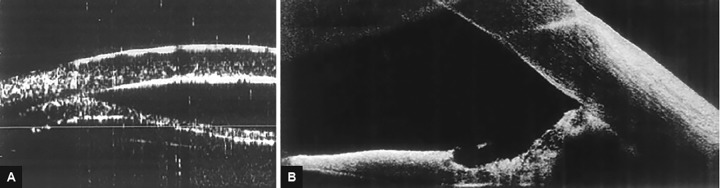
(A) Ultrasound biomicroscopy synechial angle closure in patient 5; and (B) AS-OCT synechial angle closure in patient 13. AS-OCT: Anterior segment optical coherence topography

**Figs 2A and B:**
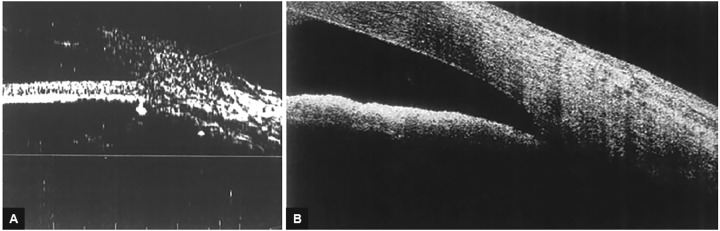
(A) Ultrasound biomicroscopy open angle in patient 22; and (B) AS-OCT showing open angle in patient 37. AS-OCT: Anterior segment optical coherence topography

One eye had a pupillary block that developed 1 week after surgery, two eyes had synechial angle closure (Fig. 1), and the rest had open-angle glaucoma (Fig. 2).

### Risk Factors for Postoperative Glaucoma

Out of the 18 eyes that developed glaucoma-related adverse event, 77.8% had their cataract surgery before the age of 12 months, 72.2% had no ocular anomalies, and none of them had systemic anomalies. Also 14 eyes (77.78%) were aphakic at the time of diagnosis, and 4 eyes (22.22%) were pseudophakic. None of those patients developed glaucoma after secondary procedure. The risk factors for glaucoma-related adverse events, concerning the age at time of cataract surgery, ocular anomalies, the state of aphakia/pseudophakia, and the axial length were studied using general estimating equation (GEE). Age ≤ 12 months at time of cataract surgery was found to be a statistically significant risk factor (p = 0.005) which means that children ≤ 12 months at time of cataract surgery are at higher risk of developing secondary postoperative glaucoma. Axial length was found to be of marginal significance (p = 0.05). On the contrary, the other parameters were found to be statistically insignificant (Table 2).

**Table 2:** **Table 2:** Risk factors for the occurrence of postoperative glaucoma, GEE and odds ratio with 95% CI

						*95 % Confidence interval*			
*Model*		*p-value*		*OR*		*Lower bound*		*Upper bound*	
Age at time of cataract surgery		0.196		0.015		–0.008		0.037	
Age at time of cataract surgery (≤12 months/>12 months)		0.005		2.049		0.618		3.481	
Ocular anomaly		0.142		–0.863		–2.013		0.288	
Aphakia/pseudophakia		0.137		–1.014		–2.349		0.321	
Axial length		0.050		–0.370		–0.741		0.001	

p-value < 0.05 is considered statistically significant

**Table 3:** **Table 3:** Risk factors for the time of occurrence of postoperative glaucoma, using Cox regression survival analysis

						*95% Confidence interval*			
*Model*		*p-value*		*Hazard ratio*		*Lower bound*		*Upper bound*	
Age at time of cataract surgery		0.377		0.987		0.959		1.016	
Age at time of cataract surgery (<12 months/>12 months)		0.099		0.392		0.129		1.192	
Ocular anomaly		0.060		2.690		0.958		7.552	
Procedure (bimanual irrigation-aspiration/lensectomy)		0.578		1.371		0.451		4.167	
Aphakia/pseudophakia		0.212		0.519		0.185		1.455	
Axial length		0.386		0.915		0.748		1.119	

p-value <0.05 is considered statistically significant

Also the effect of these factors on the time from cataract surgery till the occurrence of postoperative glaucoma-related adverse events was studied using Cox regression survival analysis, and was found to be statistically insignificant (Table 3).

## DISCUSSION

We have conducted this prospective study to better understand the incidence and risk factors of secondary glaucoma occurring within 1 year following pediatric cataract surgery in our population. We reported the incidence of postoperative glaucoma to be 11.3% (17 eyes). Variable incidences of postoperative glaucoma have been reported in other studies due to difference in study population, duration of follow- up period, parameters used for glaucoma diagnosis, inclusion and exclusion criteria.^1-4^

In the current study, the interval between cataract surgery and glaucoma adverse event diagnosis ranged from 1 week to 1 year. Surprisingly, the majority of cases (66.7%) were diagnosed in the first 3 months. This incidence is more or less comparable to the results reported by Wong et al^17^ and IATS^10^ who studied secondary glaucoma in first year following cataract surgery. The former reported that 12.2% of their patients developed glaucoma in the first year, at a mean of about 5 months,^17^ while IATS reported that 9% developed glaucoma in the first postoperative year and another 9% were diagnosed after first postoperative year (2-5 years).^10,18^

Other studies looking at glaucoma following pediatric cataract surgery reported a later onset generally, but they use criteria at IOP > 25 mm Hg^3,5^ or rely on consultants’ decision to start long-term medical and/or surgical therapy.^4^ Asrani and Wilensky^19^ noted that glaucoma was diagnosed at a mean interval of 12.2 years, with the majority of cases after the second postoperative year.

We were really puzzled by the early onset of glaucoma following cataract surgery. Mills and Robb^1^ reported that angle closure mechanism usually occurred within the first few months after surgery while open-angle glaucoma had a later onset. With introduction of modern surgical techniques, a different pattern of glaucoma has been reported where the incidence of angle closure glaucoma declined dramatically.^10,20^ This is more or less consistent with our results, where we have only one eye with pupillary block, two eyes with synechial angle closure, and the rest with open-angle glaucoma.

The age at cataract surgery and its relation to development of glaucoma is very debatable. In our study, 77.8% of those who developed glaucoma adverse events did their cataract surgery before the age of 12 months, but this was statistically insignificant. Different studies suggested different age periods to be associated with the highest risk of glaucoma.^6,21^ However, Chen et al^3^ found no particular age in the first year associated with a greater risk of aphakic glaucoma.

In our study, 14 eyes (77.78%) out of the 18 eyes that developed glaucoma adverse events were aphakic at the time of diagnosis, and 4 eyes (22.22%) were pseudopha-kic, which was statistically insignificant. There are many studies that suggested that IOL, in one way or another, is considered protective against glaucoma.^21,22^ Trivedi et al^5^ reported that glaucoma developed postoperatively in 3.8% of pseudophakic eyes and 17% of aphakic eyes, and this was statistically insignificant. Similarly, Wong et al^17^ together with IATS^10^ noted no statistically significant difference in glaucoma incidence in both the aphakic and pseudophakic groups. In our study, the age at time of cataract surgery is a determining factor for IOL implantation, as patients <1 year were routinely left aphakic. We are really unable to differentiate whether the increased rate of glaucoma in aphakic patients in our study, though it is statistically insignificant, is due to absence of IOL or the early age at surgery or both.

Microphthalmia^8,9^ and PFV^7,10^ have been documented to be important parameters for developing secondary glaucoma after cataract surgery. However, other studies failed to confirm that PFV is a risk factor.^23^ In our study, both ocular anomalies did not reach statistical significance.

The limitations of our study were the small follow-up period that may underestimate the true prevalence of glaucoma following pediatric cataract surgery, patients being operated upon by different surgeons (however, the general surgical techniques were the same), and the small sample size of glaucomatous eyes that yielded statistically insignificant results. The strength of the study is its prospective nature, and is the first study to address this issue in our population.

## CONCLUSION

It is not obvious how soon after pediatric cataract surgery secondary glaucoma may develop. This study has supported the results of other studies, where a high incidence of glaucoma was found during first year following cataract surgery. Accordingly, regular follow-up should start as early as possible.

## CLINICAL SIGNIFICANCE

Further prospective studies with larger study population are required to better understand the magnitude and risk factors for development of secondary glaucoma in our community and to establish a national regimen for follow-up of children after congenital cataract surgery.
